# A decrease in intact parathyroid hormone (iPTH) levels is associated with higher mortality in prevalent hemodialysis patients

**DOI:** 10.1371/journal.pone.0173831

**Published:** 2017-03-24

**Authors:** Ricardo Villa-Bellosta, Laura Rodriguez-Osorio, Sebastian Mas, Younes Abadi, Mercedes Rubert, Concepción de la Piedra, Carolina Gracia-Iguacel, Ignacio Mahillo, Alberto Ortiz, Jesús Egido, Emilio González-Parra

**Affiliations:** 1 Servicio de Nefrología, Fundación Jiménez Díaz, Madrid, España; 2 Fundación Instituto de Investigación Sanitaria, Fundación Jiménez Díaz (IIS-FJD), Madrid, España; 3 Spanish Biomedical Research Network in Diabetes and Associated Metabolic Disorders (CIBERDEM), Madrid, Spain; 4 Spanish Kidney Research Network (REDINREN), Madrid, Spain; 5 Servicio de Laboratorio de Bioquímica, Fundación Jiménez Díaz, Madrid, España; 6 Universidad Autónoma de Madrid, Madrid, España; Postgraduate Medical Institute, INDIA

## Abstract

**Background:**

The mortality of dialysis patients is 10- to 100-fold higher than in the general population. Baseline serum PTH levels, and more recently, changes in serum PTH levels (ΔPTH) over time, have been associated to mortality in dialysis patients.

**Methods:**

We explored the relationship between ΔPTH over 1 year with mortality over the next year in a prospective cohort of 115 prevalent hemodialysis patients from a single center that had median baseline iPTH levels within guideline recommendations.

**Results:**

Median baseline iPTH levels were 205 (116.5, 400) pg/ml. ΔiPTH between baseline and 1 year was 85.2 ± 57.1 pg/ml. During the second year of follow-up, 27 patients died. ΔiPTH was significantly higher in patients who survived (+157.30 ± 25.82 pg/ml) than in those who died (+39.03 ± 60.95 pg/ml), while baseline iPTH values were not significantly different. The highest mortality (48%) was observed in patients with a decrease in ΔiPTH (ΔiPTH quartile 1, negative ΔiPTH) and the lowest (12%) mortality in quartile 3 ΔiPTH (ΔiPTH increase 101–300 pg/ml). In a logistic regression model, ΔiPTH was associated with mortality with an odds ratio (OR) of 0.998 (95% CI 0.996–0999, p = 0.038). In multivariable analysis, mortality risk was 73% and 88% lower for patients with ΔiPTH 0–100 pg/ml and 101–300 pg/ml, respectively, than for those with a decrease in ΔiPTH. In patients with a decrease in ΔiPTH, the OR for death was 4.131 (1.515–11.27)(p = 0.006).

**Conclusions:**

In prevalent hemodialysis patients with median baseline iPTH values within the guideline recommended range, a decrease in ΔiPTH was associated with higher mortality. Further studies are required to understand the mechanisms and therapeutic implications of this observation that challenges current clinical practice.

## Introduction

Parathyroid hormone (PTH) is an 84 amino acid protein, which regulates mineral metabolism by targeting bone, kidney and intestine, among other organs. PTH is also a uremic toxin: serum levels increase progressively in the course of chronic kidney disease (CKD), and are associated to multiple systemic adverse effects, including cardiovascular disease [1–4]. However, in hemodialysis patients, PTH is a poor marker of mortality. Only large studies have identified PTH values that are associated to increased mortality, and in these studies the specific PTH values associated to mortality are highly variable [4–8]. Other molecules such as calcium, phosphorus and vitamin D have a more consistent association with mortality, although available studies are difficult to interpret [9,10].

Assessment of biomarker dynamics, i.e., changes over time, rather than the assessment at a single time point, is emerging as a potentially better indicator of outcomes. This concept has been validated for the influence of dynamic serum calcium and phosphorus levels on survival [11]. Keeping the PTH concentration within an optimal range is a key therapeutic target in CKD. This is usually achieved by prescribing drugs that lower PTH levels. Intact PTH (iPTH) levels fluctuate over time. However, few studies have addressed the potential of dynamic changes in PTH levels to predict outcomes. Recently, a relationship between changes in iPTH levels over time (ΔiPTH) and survival was observed [12]. An increase in ΔiPTH was associated with decreased mortality only in patients with low baseline iPTH levels (<168 pg/ml)[12]. The potential clinical implication, to be validated in interventional studies, is that allowing iPTH to increase when it has been oversuppressed may improve outcomes.

In this prospective study, we assessed whether ΔiPTH over one year predicts mortality in a homogeneous (single center), routine clinical practice hemodialysis population. For the first time, we have observed that in prevalent hemodialysis patients with median baseline iPTH values within the guideline recommended range, a decrease in ΔiPTH was associated with higher mortality. The potential clinical implication is that care should be exercised to prevent over suppression of iPTH levels. While in line to recent observations, the present results go a step further, raising concerns about the potential dangers of lowering iPTH levels while they remain within the recommended target.

## Materials and methods

**Fig 1:**
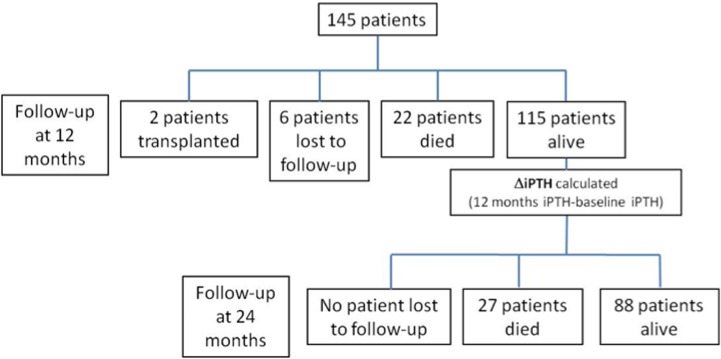
Patient flowchart.

In this prospective, observational, routine clinical practice study, 145 prevalent hemodialysis patients from a single center were monitored for changes in analytical and nutritional parameters from baseline to 12 months (first year of follow-up) and mortality was assessed during the second year of follow-up (12 to 24 months, Fig 1). The dialysate calcium concentration was 1.25 to 1.50 mmol/L in all patients. Causes of death causes were registered.

Inclusion criteria were age >18 years and dialysis vintage >6 months. Patients were excluded if they did not consent, had positive serology for HBsAg, anti-HVC or anti-HIV or a life expectancy <6 months.

Analytical parameters included serum iPTH, calcium, phosphorus, alkaline phosphatase, FGF23, 25 (OH) vitamin D, C reactive protein (CRP), total protein and albumin. iPTH values were determined by a second generation electrochemiluminescence method in an Elecsys autoanalyzer (Roche), using an antibody directed against amino acids 26–32 and another against amino acids 55–64. This assay does not detect short carboxy-terminal fragments, but identifies PTH 1–84 and long 7–84 fragments and amino PTH [13]. Intra- and inter-assay coefficients of variation were <2.5% and <3% respectively, with a limit of detection of 1.2 pg/ml. iPTH values were normalized according to the Spanish Society of Nephrology (SEN) equation [13]. Total 25 (OH) vitamin D (D2 and D3) was determined by electrochemiluminescence in an Elecsys autoanalyzer (Roche) with intra- and inter-assay coefficients of variation of <7.5% and <8%, respectively and a limit of detection of 3 ng/ml. Serum albumin, calcium, creatinine, inorganic phosphorus, total protein, CRP and alkaline phosphatase were determined in an ADVIA CENTAUR 2400 autoanalyzer, following the manufacturer protocols. Plasma Fibroblast Growth Factor 23 (FGF-23, C-terminal) was determined by ELISA (Immutopics, USA) using two polyclonal antibodies directed to the C-terminus of FGF-23 (intra-assay and inter-assay coefficients of variation <1.7% and <3.5%, sensitivity 1.5 RU / ml).

### Ethics statement

The study protocol was approved by the Institutional Review Board and Ethics committee of the Jimenez Díaz Foundation (Ref. 2016/15). Clinical research followed the principles of the Declaration of Helsinki and Spanish Law. Participans provide a written consent to participate in this study.

### Statistical analysis

Quantitative data are presented as median (interquartile range), or mean ± standard deviation. The level of significance was set at p<0.05. Qualitative variables are presented as percentages. Differences in baseline characteristics were assessed using χ2 or Fisher´s exact test when appropriate for qualitative data and Student t test for quantitative data. Distribution of data was tested using the Shapiro-Wilk normality test. Percent changes from baseline measurements were analyzed by Wilcoxon signed-rank test. Differences between iPTH values at baseline and at 1 year were assessed using Mann-Whitney test. Associations between different variables were evaluated by Spearman correlation. Analyses were performed with IBM SPSS Statistics for Windows, Version 20 (Armonk, NY: IBM Corp).

## Results

### A decrease in ΔiPTH levels within 12 months is associated with increased mortality in the next 12 months

Table 1 shows the general characteristics of the study population. During the first year of follow-up, 22 patients died, 6 patients were lost to follow-up and 2 patients were transplanted (**Fig 1**). Thus, the change in iPTH levels (ΔiPTH) between baseline and 1 year was calculated in 115 patients (Table 2). Median baseline iPTH levels were 205 (116.5, 400) pg/ml, within the guideline recommended range. During the first year of follow-up, ΔiPTH was positive (85.2 ± 57.1 pg/ml). That is, a mean increase in ΔiPTH was observed from baseline to 1 year.

**Table 1:** Demographic and clinical characteristics of the study population. Data expressed as mean SD, median (interquartile range) or %.

Age (years)	65.9 ± 14.6
Dry weight (Kg)	65.5 ± 14.2
Dialysis vintage (years)	3 (2, 6)
Male sex (%)	51
Kt/v urea	1.79
Vascular access (%)	
*fistula	60.8
*graft	19.2
* catheter	20
Hemodialysis modality (%)	
*Conventional	90
* On-Line hemodiafiltration	10
Hypertension (%)	86.9
Diabetes mellitus (%)	23.4
Dyslipidemia (%)	35.2

**Table 2:** Baseline, first year (12 months) and second year (24 months) analytical values. Data expressed as mean ±SD or median (interquartile range).

Variable	Baseline (N = 115)	First year (N = 115)	Second year (N = 88)
Hemoglobin (g/dl)	11.9 ± 1.64	11.9 ± 1.2	11.4 ± 1.2
Hematocrit (%)	35.7 ± 4.54	37.3 ± 3.8	36.5 ± 3.8
Albumin (g/dl)	3.70 ± 0.47	3.7 ± 0.4	3.8 ± 0.3
Calcium (mg/dl)	9.15 ± 0.72	9.0 ± 0.6	10.1 ± 0.8
Phosphorus (mg/dl)	5.53 ± 9.07	4.5 ± 1.4	4.6 ± 1.5
Total CO2 (mEq/L)	20.92 ± 3.47	24.1 ± 3.4	24.7 ± 2.6
Ferritin (ng/dl)	401.9 ± 242.4	614 ± 410	494 ± 337
iPTH (pg/ml)	205 (116.5, 400)	290 (139, 539)	310 (153, 517)
25OH-vitamin D (ng/dl)	22.4 (12.7,34.2)	26.8 (16.7, 38.7)	28.1 (19.1, 37.7)
Creatinine (mg/dl)	7.95 (5.6,9.1)	7.4 (6.2, 9.0)	7.5 (6.5, 8.7)
Alkaline phosphatase (UI/l)	110.0 (89,145)	102 (78, 133)	99 (73, 129)
C-reactive protein (mg/dl)	0.6 (0.25,1.97)	1.6 (0.5, 3.2)	1.4 (0.7, 3.6)

**Fig 2:**
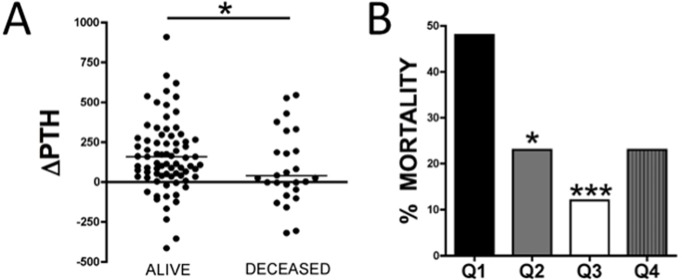
Changes in iPTH (ΔiPTH,pg/ml) during the first 12 months and mortality at 24 months. (A) Comparison of ΔiPTH within the first 12 months between survivors and deceased patients at 24 months. The mean ΔiPTH was higher in survivors than in deceased patients. * P <0.*05*. (B) Graphic representation of mortality according to quartiles of ΔiPTH in the first year: Q1 ΔiPTH <0 pg/ml; Q2 0–100 pg/ml; Q3 101–300 pg/ml; Q4 >301 pg/ml. * P <0.05 *** p <0.*001 vs Q1*.

We compared ΔiPTH during the first year in patients who survived the second year of follow-up (n = 88 alive at 24 months of follow-up) with those in patients who died during the second year of follow-up (n = 27). Causes of death were as follows: 20 patients died from cardiovascular disease, 4 from infection, 1 from cancer and 2 from wasting. The increase in ΔiPTH was significantly higher in patients who survived (+157.30 ± 25.82 pg/ml) than in those who died (+39.03 ± 60.95 pg/ml, p <0.05) (Fig 2A). By contrast, there were no significant differences in baseline iPTH between survivors and non-survivors [199.9 pg/ml (118.6–338.1) vs 231.0 (102.3–508.2) pg/ml, ns] and in the percentage of patients with low baseline iPTH levels between survivors, (21/88, 23.9%) and non-survivors (13/27, 48.1%). Baseline iPTH was <150 pg/ml in 7/26 (27%) patients with a decrease in ΔiPTH and in 27/89 (30%) patients with an increase in ΔiPTH. Thus, there were also no differences in the percentage of patients with low baseline iPTH levels between the two ΔiPTH categories.

When patients were divided into quartiles of ΔiPTH within the first year, the highest mortality (46%) was found in patients with a decrease in ΔiPTH (ΔiPTH <0 pg/ml, quartile 1, Fig 2B). In quartile 2 (ΔiPTH increase 1–100 pg/ml) and quartile 3 (ΔiPTH increase 101–300 pg/ml) the overall mortality (23 and 12%, respectively) was significantly lower than quartile 1. Thus, the lowest mortality was observed in quartile 3 of ΔiPTH (ΔiPTH increase between 101 and 300 pg/ml). ΔiPTH seems therefore a good marker of survival during the study period.

In a large pan-European study, the lowest mortality risk was observed in hemodialysis patients with serum PTH values 168–674 pg/mL and increases in serum PTH in patients with baseline values <168 pg/mL were associated with improved survival [12], so we addressed whether the association of ΔiPTH with mortality was independent from baseline iPTH levels. Subgroup analysis did not disclose significant differences in baseline iPTH. However, the low number of patients in each subgroup precludes obtaining any definitive conclusions. Thus, 34/115 patients had a baseline iPTH <150 pg/ml, 42/115 had iPTH 150–300 pg/ml and 39/115 had iPTH >300 pg/ml, of which only 11 had iPTH> 600 pg/ml. Among the 89 patients with ΔiPTH ≥0 pg/ml at 1 year, the number of deaths was 8/27 (29.6%) for baseline iPTH <150 pg/ml, 2/40 (5%) for baseline iPTH 150–300 and 5/22 (22.7%) for baseline iPTH> 300 pg/ml.

Few significant differences were observed in baseline clinical and laboratory characteristics between patients with positive or negative ΔiPTH (Table 3 and Table 4). Baseline serum calcium was significantly lower and any vitamin D treatment significantly higher in patients with a decrease in ΔiPTH. The lower serum calcium did not appear to be associated with calcimimetic use, since no differences were observed between patients with an increase or a decrease in ΔiPTH.

**Table 3:** Baseline quantitative variables in patients with or without a decrease in ΔiPTH during the first year. Data expressed as Mean ±SD.

ΔiPTH	<0 pg/ml (n = 26)	≥0 pg/ml (n = 89)	P value
Age (years)	65.6 ± 13.9	66.0 ± 14.6	0.888
Dialysis vintage (years)	2.00 ± 4.00	3.00 ± 4.00	0.190
EPO dose (IU/week)	6000 ± 8000	6000 ± 6000	0.762
Calcium (mg/dl)	8.84 ± 0.66	9.20 ± 0.73	**0.029**
Phosphorus (mg/dl)	5.15 ± 1.70	5.85 ± 11.04	0.587
Hemoglobin (g/dl)	12.0 ± 1.35	11.8 ± 1.72	0.528
FGF-23 (RU/ml)	747 ± 1918	666 ± 1749	0.736
Alkaline phosphatase (UI/l)	113.5 ± 41.0	110.0 ± 57.5	0.672
C-reactive protein (mg/dl)	0.25 ± 1.15	0.33 ± 1.60	0.568
25OH-vitamin D (ng/dl)	21.1 ± 20.0	22.4 ± 21.4	0.961
Albumin (g/dl)	3.62 ± 0.38	3.67 ± 0.46	0.617

**Table 4:** Baseline qualitative variables (gender and medication) for patients with or without a decrease in ΔiPTH during the first year. Data expressed as n (%).

DiPTH	<0 pg/ml (n = 26)	≥0 pg/ml (n = 89)	P value
Male Sex	13 (50.0)	41 (46.1)	1.000
25OH vitamin D therapy	10 (38.5)	27 (30.3)	0.977
Calcitriol therapy	4 (15.4)	7 (7.9)	0.464
Paricalcitol therapy	8 (30.1)	19 (21.3)	0.751
Any vitamin D therapy *	22 (84.6)	53 (66.8)	**0.033**
Cinacalcet	3 (11.5)	12 (13.4)	0.754
Calcium-based phosphate binder	10 (38.5)	28 (31.4)	1.000
Sevelamer	10 (38.5)	26 (29.2)	0.879

*25OH vitamin D or calcitriol or paricalcitol

### Small increments in ΔiPTH(<300 pg/ml) were associated with better survival

In univariate analysis logistic regression modelling, increasing age, lower baseline albumin and a decrease in ΔiPTH were predictors of mortality. ΔiPTH within 1 year was associated with mortality (dependent variable) with an odds ratio of 0.998 (95% CI 0.996–0999, p 0.038)(Table 4). In addition, an increased (as opposed to a decreased ΔiPTH) as well as an increased ΔiPTH in the 100–300 pg/ml range were associated with lower mortality (Table 5).

**Table 5:** Univariate logistic regression models for predicting mortality. Higher baseline age, lower baseline serum albumin and a decreasing ΔiPTH (ΔiPTH<0 pg/ml) within the first year are predictors of mortality.

Variable	OR (95% CI)	P value
Baseline age (years)	1.062 (1.030–1.096)	<0.001
Female Sex	0.607 (0.301–1.225)	0.162
Dialysis vintage (years)	1.009 (0.942–1.080)	0.806
Baseline PTH (pg/ml)	1.001 (1.000–1.002)	0.071
Baseline FGF23 (RU/ml)	1.000 (1.000–1.000)	0.327
Baseline calcium (mg/dl)	1.260 (0.792–2.002)	0.328
Baseline phosphate (mg/dl)	0.974 (0.888–1.068)	0.424
Baseline 25(OH)Vit D (ng/dl)	0.996 (0.987–1.005)	0.321
Baseline albumin (g/dl)	0.379 (0.171–0.843)	0.013
ΔiPTH (continuous variable in pg/ml)	0.998 (0.996–1.000)	0.037
ΔiPTH binary categories		
ΔiPTH<0 pg/ml	1.000	
ΔiPTH≥0 pg/ml	0.262 (0.100–0.689)	0.007
ΔiPTH quartiles (pg/ml)		
Q1 <0	1.000	
Q2 0–100	0.310 (0.093–1.027)	0.055
Q3 100–300	0.120 (0.029–0.501)	0.004
Q4 >300	0.464 (0.135–1.600)	0.224

In a step-by-step multivariable models built with all variables with p values <0.25 in univariate models, and considering increasing age and lower baseline albumin as potential confounders, the variables most strongly related with mortality were age (in one of the models) and ΔiPTH, assessed as either a binary categorical variable (increase/decrease) or as quartiles (Table 6). Patients with an increase in ΔiPTH had a statistically significant 76% lower mortality risk than those with a decrease in ΔiPTH (negative ΔiPTH). Additionally, patients with a ΔiPTH between 0 and 100 pg/ml had a statistically significant 73% lower mortality risk than those with a decrease in ΔiPTH (negative ΔiPTH)(Table 6). Patients with ΔiPTH 101–300 pg/ml had a statistically significant 88% lower mortality than those with a decrease in ΔiPTH. However, a ΔiPTH increase above 301 pg/ml was not associated with a survival advantage when compared with those with a decrease in ΔiPTH. The predictive ability of the model according to ROC curve analysis is acceptable, with an area under the curve of 0.74. A decrease in ΔiPTH was associated with an OR for death of 4.131 (1.515–11.27) with a p = 0.006. When ΔiPTH was considered a continuous variable, the multivariable model was consistent with results obtained when comparing a decrease versus an increase in ΔiPTH, and there was a trend towards lower mortality associated with higher ΔiPTH (2%_o_ lower with every 1 pg/ml increase in ΔiPTH, Table 7).

**Table 6:** Multivariable regression model for mortality prediction considering ΔiPTH within the first year as a binary variable (decrease/increase) or as quartiles.

Model	Variable	OR (95% CI)	P value
Model 1	ΔiPTH (pg/ml)		
	Decrease	1.000	Reference
	Increase	0.242 (0.089–0.660)	0.006
	Age	1.040 (1.000–1.080)	0.047
	Albumin	0.951 (0.314–2.882)	0.929
Model 2	ΔiPTH (pg/ml)		
	Q1 (<0)	1.000	Reference
	Q2 (0–100)	0.264 (0.076–0.922)	0.037
	Q3 (100–300)	0.115 (0.027–0.490)	0.003
	Q4 (>300)	0.475 (0.131–1.717)	0.256
	Age	1.039 (1.000–1.079)	0.049
	Albumin	0.893 (0.286–2.776)	0.846

**Table 7:** Multivariable regression model for mortality prediction considering ΔiPTH within the first year as a continuous variable.

Model	Variable	OR (95% CI)	P value
Model 1	ΔiPTH (pg/ml)	0.998 (0.996–1.000)	0.058
	Age	1.035 (0.997–1.074)	0.068
	Albumin	0.904 (0.311–2.625)	0.852

## Discussion

The main finding of the present report is that ΔiPTH within the first year of follow-up is a marker of mortality during the second year of follow-up in patients on hemodialysis with median baseline iPTH values within the recommended range. Thus, a decrease in ΔiPTH was associated with higher mortality even after multivariate adjustment, and a mild increase in ΔiPTH (101–300 pg/ml) was associated with the lowest mortality.

Optimal target iPTH values in hemodialysis were established by bone histo-morphometric criteria [14,15] and by the association of iPTH levels with overall mortality [16]. However, published results on the relationship between mortality and baseline iPTH values are very heterogeneous. The vast majority of studies addressing this relationship were performed assessing baseline iPTH levels and time-fixed survival. However, as other biological variables, iPTH levels are dynamic and change over time. Longitudinal changes in serum calcium, phosphate and calcium x phosphate product have been associated with mortality [11]. In this regard, a decrease in iPTH over 3 months was associated with a higher risk of all-cause mortality only in patients with a concurrent decrease in body mass index (BMI) [17]. However, ΔiPTH was likely a surrogate marker for changes in BMI, and the association to all-cause mortality was explained by protein energy wasting [17].

In a further study, changes in serum iPTH were assessed as categorical variables. A decrease from baseline high-normal iPTH levels to low levels (heterogeneous definition: <2x lower than the upper limit of normal of the iPTH assay at each center) during the first year of hemodialysis in incident patients, was an independent and strong risk factor for cardiovascular death in the following year, but not for all-cause death, (20). However, the main contributor to the observed decrease in serum iPTH was the use of a high-calcium dialysate concentration (1.75 mmol/L) which was also independently associated with higher mortality risk [18]. By contrast, patients with low baseline iPTH levels that remained within the low iPTH range at month 12 had no increase in cardiovascular death risk and it was not studied whether a decrease in iPTH remaining within the normal-high range was associated with mortality. Our study explored a different research questions, the prognostic value of ΔiPTH, independently of baseline iPTH levels or of whether a change in iPTH category occurred, using ΔiPTH as either a continuous variable or a categorical variable in a homogenous population from the point of view of the iPTH assay used and of dialysate calcium. Furthermore, given that a high dialysate calcium (1.75 mml/L) was not prescribed, this was not a factor in our study.

The pan-European COSMOS study findings were in line with the present findings [12], although the approach and clinical implications were different. COSMOS observed a lower mortality in patients with baseline iPTH levels between 168 and 674 pg/mL. Furthermore, in patients with low baseline iPTH levels (<168 pg/ml), but not in those with baseline iPTH >168 pg/ml, an increase in ΔiPTH was associated with decreased mortality. To our knowledge, our study is the first to describe than in a hemodialysis population with median iPTH values within the guideline recommended range, a decrease in ΔiPTH is associated with higher mortality. It is likely that the high heterogeneity in the COSMOS study regarding patient characteristics and therapeutic approaches, inherent to multicenter, multinational studies, may underlie the failure to observe an association between a decrease in ΔiPTH and mortality. In this regard, in line with our findings, a non-significant trend was observed towards higher mortality when ΔiPTH decreased which was more apparent for patients with low baseline iPTH. Our single center study encompassed patients treated from the predialysis stage in more homogenous manner and this may have facilitated the observation of significant differences in mortality, according to ΔiPTH in patients with mean baseline iPTH levels within the target range.

A further study observed a significantly lower mortality only in patients with baseline iPTH <150 pg/ml that experienced an increase to 150–300 pg/ml [9]. However, the mortality risk did not differ between patients who maintained a high serum iPTH level (≥300 pg/mL) and those with a decrease in iPTH levels from ≥300 pg/mL to 150–300 pg/mL.

The impact of ΔiPTH during the first year on mortality shows a J-shaped form. A mild increase in ΔiPTH (<300 pg/ml) seems to be associated with lower mortality, although this association was not significant in patients with higher ΔiPTH. In this regard, as this was a routine clinical practice observational study, the number of patients with high baseline iPTH values was too low for any meaningful conclusions. The mechanisms underlying the relationship between ΔiPTH and mortality requires further investigation, as it has the potential to modify daily clinical practice. Possible explanations include the use of therapies that lower iPTH, especially those that have been reported to associate with a negative influence on survival, as may be the case for high dialysate calcium [18]or the presence of nutrition abnormalities such as malnutrition or obesity. However, no data supported these associations in our population, although the number of patients was too low to obtain meaningful information in this regard.

This observational study cannot establish a cause-effect relationship, but it raises an interesting hypothesis with broad clinical relevance: in the current clinical practice context, decreasing iPTH may be less safe than letting iPTH increase. Since a decrease in iPTH is usually the result of a therapeutic intervention, these results suggest that the current approach to treat hyperparathyroidism should be reevaluated with a focus on the potential adverse impact on mortality of a progressive suppression of iPTH levels. There is little information from observational studies or clinical trials on the relationship between the magnitude of ΔiPTH in response to PTH lowering therapy and mortality. Prescription of drugs used to lower serum iPTH did not generally correlate with higher cardiovascular and all-cause mortality in randomized trials [19]. In our study, patients with a decrease in ΔiPTH were treated with any vitamin D treatment more often than the other ΔiPTH groups. However, in observational studies, use of vitamin D receptor agonists was associated with higher survival, even when iPTH was <150 pg/ml [20,21]. Higher dialysate calcium contributes to lower iPTH but also favors a positive calcium balance that has been associated with vascular calcification and mortality. This is not the case in our study, as dialysate calcium concentration was 1.25 to 1.5 mmol/L in all patients. The use of calcium-containing phosphate binders may also cause a positive calcium balance and lower iPTH levels. In a recent meta-analysis, all-cause mortality was higher in patients randomized to calcium-based phosphate binders than to calcium-free binders in clinical trials [22]. However, in our study no differences in phosphate binder type was observed between patients with positive or negative ΔiPTH.

Nutritional status may be a confounder since it modulates PTH levels and both malnutrition and BMI have been associated to mortality in dialysis patients. Low serum albumin levels have been associated with a decrease in serum iPTH [23], while obesity was associated to higher PTH levels in the general population [24]and in pre-dialysis patients [25]. PTH is associated with BMI and with BMI longitudinal changes in dialysis patients [23]. However, in the present study no differences were observed in serum albumin between patients with positive or negative ΔiPTH.

Our study has several weaknesses. It is an observational study and cause and effect cannot be explored. Furthermore, the number of patients is low and being a single center study, the result cannot be extrapolated to other centers of countries. However, the homogeneity in dialysis initiation and treatment decision criteria may have contributed to the observation of differences that may be lost in multinational or multicenter studies.

In conclusion, dynamic changes of iPTH over time (ΔiPTH) may have prognostic value that differs from cross-sectional iPTH levels. Further studies are required to understand the mechanisms underlying the observed association between a decrease in ΔiPTH and mortality when median baseline iPTH levels are within the guideline recommended range. This information complements previous studies with the novel information any decrease in ΔiPTH is associated with higher mortality. The observation is hypothesis-generating and clinical trials designed to validate the hypothesis may modify current therapeutic approaches to secondary hyperparathyroidism in order to optimize survival.

## Supporting information

S1 Table Individual data point. (XLSX)
